# Data for evolutive analysis of insulin related peptides in bilaterian species

**DOI:** 10.1016/j.dib.2018.12.050

**Published:** 2018-12-18

**Authors:** Maëva Cherif--Feildel, Clothilde Heude Berthelin, Guillaume Rivière, Pascal Favrel, Kristell Kellner

**Affiliations:** Normandy University, Caen, France. University of Caen Normandie, Unity Biology of Organisms and Aquatic Ecosystems (BOREA), MNHN, Sorbonne University, UCN, CNRS, IRD, Esplanade de la Paix, 14032 Caen, France

## Abstract

In bilaterian species, the amino acid sequence conservation between Insulin related peptides is relatively low except for the cysteine residues involved in the disulphide bonds. In the A chain, the conserved cystein residues are included in a signature motif. Investigating the variations in this motif would give insight into the phylogenetic history of the family. The table presented in this paper contains a large set of insulin-related peptides in bilateral phylogenetic groups (deuterostomian, ecdysozoan, lophotrochozoan). NCBI databases *in silico* wide screening combined with bibliographic researches provided a framework for identifying and categorising the structural characteristics of these insulin related peptides. The dataset includes NCBI IDs of each sequence with hyperlinks to FASTA format. Moreover, the structural type (α, β or γ), the A chain motif, the total number of cysteins, the C peptide cleavage mode and the potential additional domains (D or E) are specified for each sequence. The data are associated with the research article “Molecular evolution and functional characterisation of insulin-related peptides in molluscs: contributions of *Crassostrea gigas* genomic and transcriptomic-wide screening” [1]. The table presented here can be found at http://dx.doi.org/10.17632/w4gr8zcpk5.4#file-21c0f6a5-a3e3-4a15-86e0-e5a696458866.

**Specifications table**

**Table:** 

Subject area	*Biology*
More specific subject area	*Insulin*
Type of data	*Table (online data)*
How data was acquired	*Screening in NCBI׳s databases*
Data format	*Analysed data*
Experimental factors	*In silico wide screening and bibliographic researches*
Experimental features	*Research of insulin related peptides was done in NCBI׳s databases and identification of each domain was determined according to insulin structural characteristics and bibliography.*
Data source location	*Data were collected on NCBI and in cited bibliography.*
Data accessibility	Data is available via the following link:
	http://dx.doi.org/10.17632/w4gr8zcpk5.4#file-21c0f6a5-a3e3-4a15-86e0-e5a696458866
Related research article	Cherif--Feildel, M., Heude Berthelin, C., Adeline, B., Rivière, G., Favrel, P., Kellner, K., 2018. Molecular evolution and functional characterisation of Insulin Related Peptides in molluscs: contributions of Crassostrea gigas genomic and transcriptomic-wide screening. Gen. Comp. Endocrinol. 0–1. https://doi.org/10.1016/j.ygcen.2018.10.019[1]

**Data value**

• These data provide a database for the structural characteristics of a large set of insulin related peptides in deuterostomian, ecdysozoan and lophotrochozoan species.
• The data may be used to investigate the conservation of structural motifs in bilaterian insulin related peptides.
• The data could give a basis for further phylogenetic analyses.

## 1 Data

The dataset provides a list of hyperlinks to insulin-related peptides NCBI ID originating from various metazoan species (referenced as [1], [2], [3], [4], [5], [6], [7], [8], [9], [10], [11], [12], [13], [14], [15], [16], [17], [18], [19], [20], [21], [22], [23], [24], [25], [26], [27], [28], [29], [30], [31], [32], [33], [34], [35], [36], [37], [38], [39], [40], [41], [42], [43], [44], [45], [46], [47], [48], [49]). For each sequence, the key motif of the A chain associated to the number and position of disulphide bonds was specified. The structural type, the total number of cysteins, the cleavage of C peptide and the possible additional domains were also indicated. The data is available at http://dx.doi.org/10.17632/w4gr8zcpk5.4#file-21c0f6a5-a3e3-4a15-86e0-e5a696458866.

## 2 Experimental design, materials, and methods

All sequences were identified either using wide screening of NCBI sequence databases or on the basis of bibliographic analysis (for NCBI unreferenced sequences). Clustal Omega (http://www.ebi.ac.uk/Tools/msa/clustalo/) was used to help identify domains, cleavage sites and conserved cysteins. The structural type α, β or γ was determined according to Matsunaga et al. [2] for *Caenorhabditis elegans* insulin-related peptides. The C peptide cleavage mode and the presence of additional domains D (uncleaved) or E (cleaved) resulted from this information. The variations in the A chain signature motif characterising the family [50] were specified for each sequence.
